# Supplementary figures and images for: To Correct or Not Correct? Actual Evidence, Controversy and the Questions That Remain Open
Source: J Clin Med. 2020 Jun 24;9(6):1975. doi: 10.3390/jcm9061975 (PMC7356996; doi:10.3390/jcm9061975)

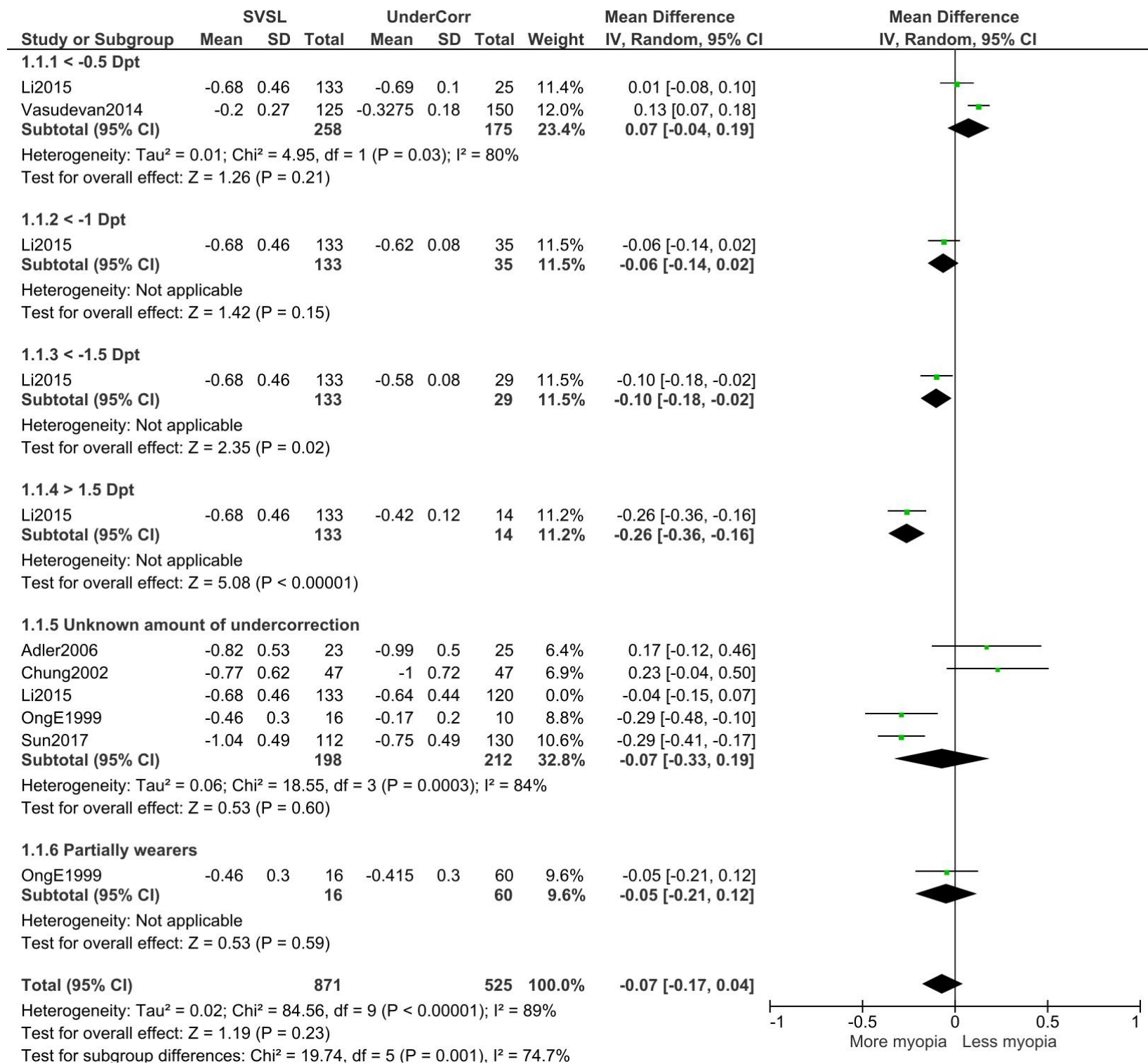

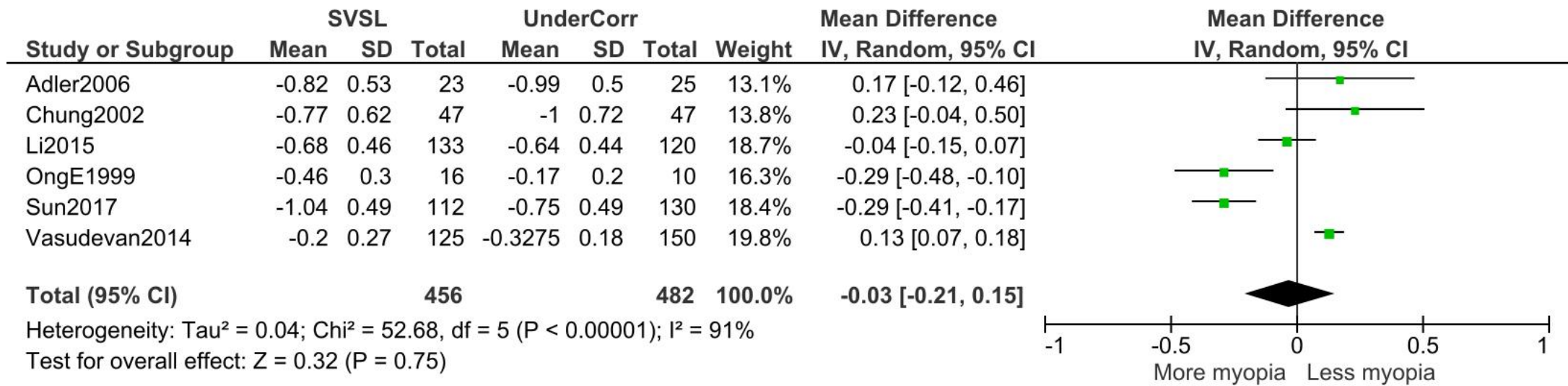

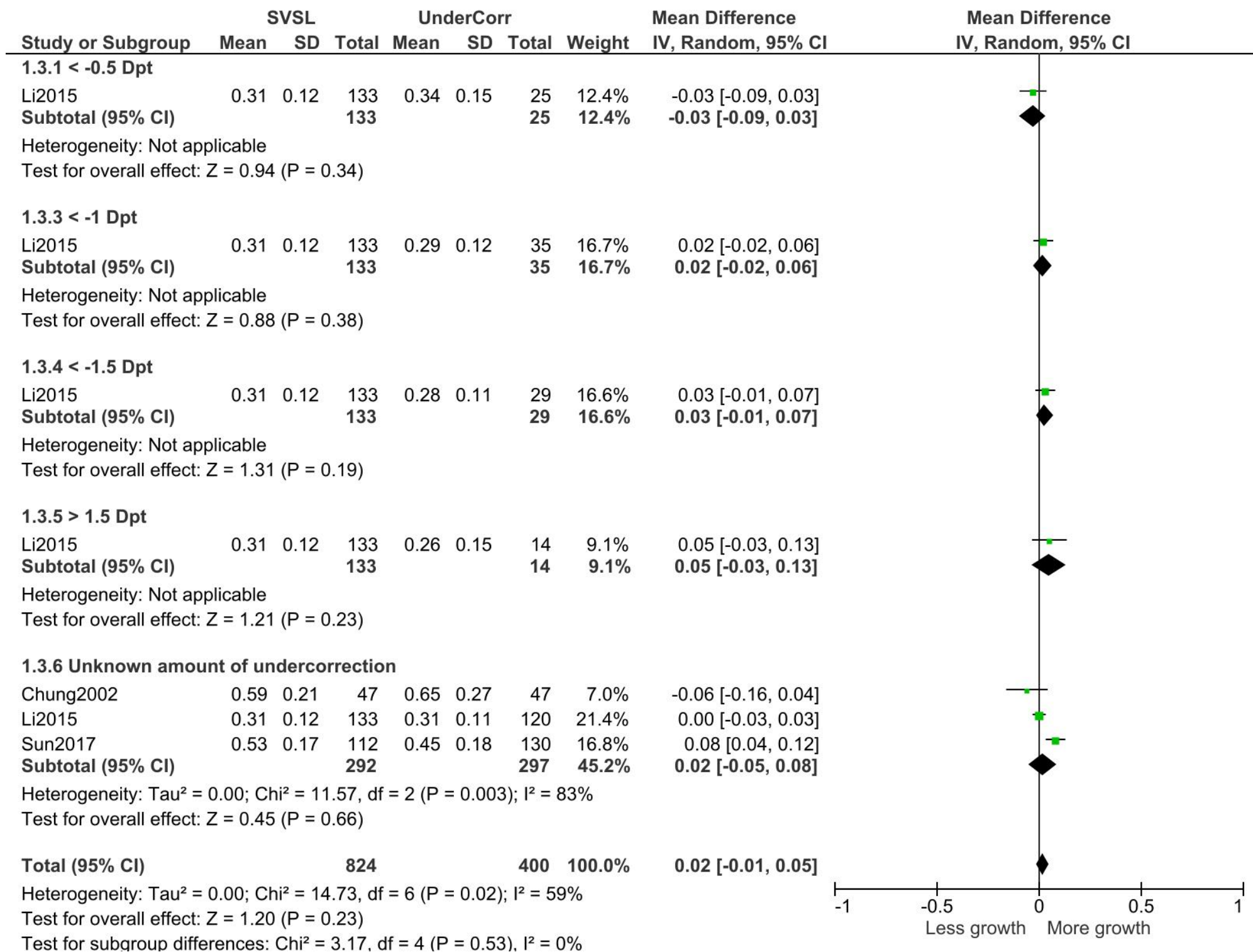

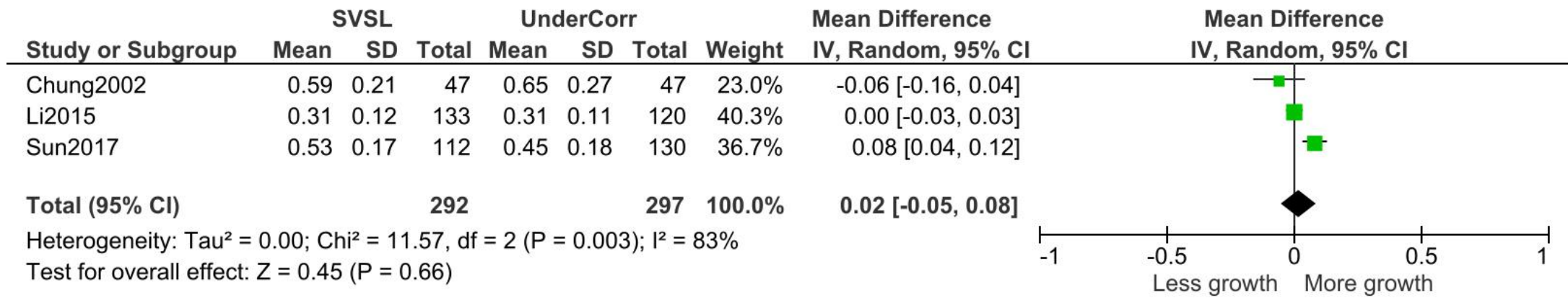

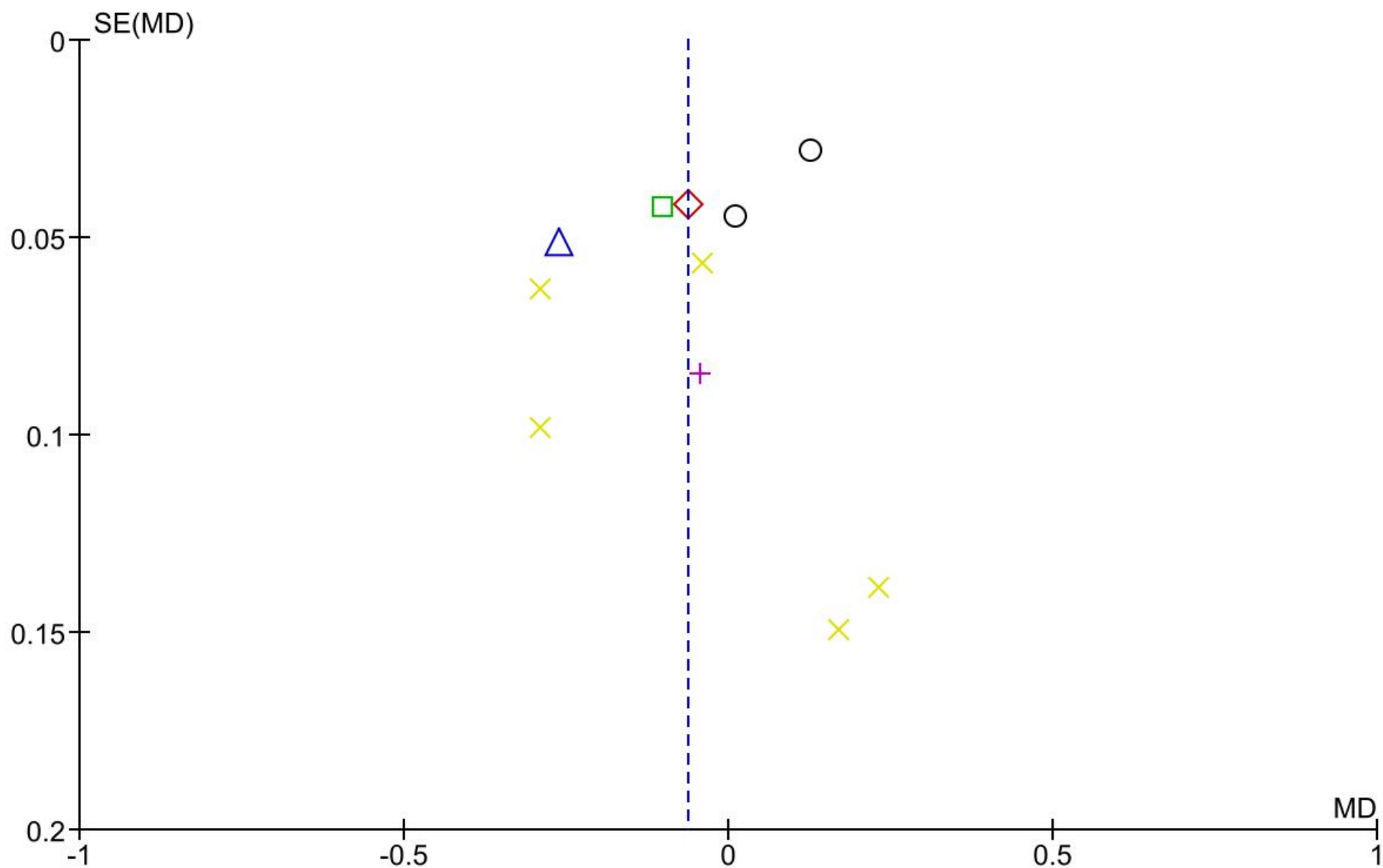

**Subgroups**

- < -0.5 Dpt
- ◇ < -1 Dpt
- < -1.5 Dpt
- △ > 1.5 Dpt
- × Unknown amount of undercorrection
- + Partially wearers

Supplement: Supplementary file 1 [file jcm-09-01975-s001.pdf]
